# Williamson County Medical Society

**Published:** 1886-02

**Authors:** 


### SOCIETY NOTES.

### WILLIAMSON COUNTY MEDICAL ASSOCIATION.

On the 13th January ult., there was a gathering of the regular physicians of Williamson county, at the Odd Fellows' Hall, in Georgetown, on which occasion an organization was effected. A Constitution and By-Laws were adopted, and the following officers were elected, to-wit: President, Dr. J. E. Walker (recognized as the "Nestor" of medicine in Williamson); Vice Presidents, Drs. H. H. Thorp, Liberty Hill, and W. M. Burgen, of Granger; Secretary, Dr. T. W. Jones, of Georgetown; Treasurer, Dr. E. W. Walton, also of Georgetown.

Some twenty members signed the Constitution, thus coming into the fold,—in accord with, and subject to the restraints and the benefits of the State Association. We were present by invitation, and also Dr. W. J. Burt—the well known Secretary of the Texas State Association. He and "we" beg here to tender our acknowledgments for the courtesies extended to us on all hands. It was an occasion long to be remembered, by us, at least. At night there was a banquet, in honor of the occasion, at which there was the usual "flow" and "feast;" speech-making, toasts and responses, anecdotes and reminiscences were the order, and characterized by the utmost good fellowship and hilarity,—*genuine*, (no wine),—hilarity; the hours flew with flying feet, *et cetera.* We sincerely trust the example will be followed in other counties, till throughout the length and breadth of this favored land, there shall resound the note of reform and organization, and every county, thoroughly organized, shall be able to send up its brightest medical minds to enrich, still further, the Convention of our glorious State Association!

