# Erratum

**Published:** 1886-02

**Authors:** 


☞ ERRATUM.—Page 370, 9th line from bottom, for "purifaction" read "purification;" (and if you find any more mistakes, correct them !)

